# Determination of inorganic phosphorus in serum: Evaluation of three methods applied to the Technicon RA-1000 analyzer

**DOI:** 10.1155/S1463924689000350

**Published:** 1989

**Authors:** L. Rubino, V. Catapano, G. Guerra

**Affiliations:** Clinical Chemistry Laboratory ‘Borselli’ Hospital (USL 31) Bondeno Ferrara 44012 Italy

## Abstract

We have evaluated three analytical methods for determining inorganic phosphorus in serum applied to the Technicon RA-I000 analyzer: a fully enzymatic colorimetric method based on the specific system purine nucleoside phosphorylase/xanthine oxidase coupled to an indicator colorimetric reaction similar to the Trinder reaction; a chemical method involving the direct UV measurement of the phosphomolybdate complex; and a chemical method with reduction of the phosphomolybdate complex to molybdenum blue. Experiments were performed to assess within-run and between-day precision, linearity, interference and correlation. The best performance characteristics were shown by the enzymatic colorimetric method and the phosphomolybdate UV method.


Journal of Automatic Chemistry Vol. 11, No. 4 (July–August 1989), pp. 164–167

### Determination of inorganic phosphorus in serum: Evaluation of three methods applied to the Technicon RA-1000 analyzer

**L. Rubino, V. Catapano and G. Guerra**
*Clinical Chemistry Laboratory, 'Borselli' Hospital (USL 31), 44012 Bondeno, Ferrara, Italy*

*We have evaluated three analytical methods for determining inorganic phosphorus in serum applied to the Technicon RA-1000 analyzer: a fully enzymatic colorimetric method based on the specific system purine nucleoside phosphorylase/xanthine oxidase coupled to an indicator colorimetric reaction similar to the Trinder reaction; a chemical method involving the direct UV measurement of the phosphomolybdate complex; and a chemical method with reduction of the phosphomolybdate complex to molybdenum blue. Experiments were performed to assess within-run and between-day precision, linearity, interference and correlation. The best performance characteristics were shown by the enzymatic colorimetric method and the phosphomolybdate UV method.*

### Introduction

Phosphorus and phosphorylated compounds are largely involved in the main biochemical process, that is bone, carbohydrate, lipid, nucleic acids and energy metabolism. Inorganic phosphorus is the fraction commonly determined in the clinical laboratory and the clinical significance of the measurement of inorganic phosphorus in serum and urine is widely recognized [1,2].

Various analytical techniques have been employed for determining inorganic phosphorus in serum, but the most widely used in the routine work is photometry. Many chemical methods, based on ultraviolet or colorimetric measurements, have been described, however, the continuous proposal of new methods attests a certain dissatisfaction for those available. Enzymatic methods have been described based on UV measurement [3–6] and on colorimetry [7]. Recently, an enzymatic colorimetric method has been made available in a kit format and has gained wide acceptance for its performance characteristics and convenience [8–10].

In the work described in this paper we compared this enzymatic colorimetric method with a chemical method based on the direct UV measurement, and a colorimetric method based on the chemical reduction, all applied to a Technicon RA-1000 analyzer.

### Materials and methods

We applied the following methods to a Technicon RA-1000 random-access analyzer (Technicon Instruments Corp., Tarrytown, N.Y. 10591, USA), carrying out all assays at 37 °C and calibrating the instrument before linearity, precision and correlation experiments with an aqueous standard at 1·29 mmol/l of inorganic phosphorus (Chemetron Chimica SpA, Milan, Italy).

*Enzymatic colorimetric method*

Inorganic phosphates react first with inosine, in the presence of purine nucleoside phosphorylase (EC 2.4.2.1), to form ribose-1-phosphate and hypoxanthine. The latter is then oxidised by xanthine oxidase (EC 1.1.3.22) to hydrogen peroxide and xanthine, which is in turn oxidised to uric acid with further production of hydrogen peroxide. The liberated hydrogen peroxide reacts, in the presence of peroxidase (EC 1.11.1.7), with the chromogen system 4-aminophenazone/*N*-ethyl-*N*-(3-methylphenyl)-*N'*-acetylethylenediamine with the formation of a red–purple chromogen quinonimine which has an absorption peak at 550 nm [8–10].

The reagent kit used was from Miles Italiana SpA, Divisione Diagnostici, Cavenago Brianza, Italy. Reagents were reconstituted as per manufacturer's instructions and a single working solution was prepared. The instrument settings were as follows:

| | |
|---|---|
| CHEM # | |
| NAME | 11 I.PH |
| TYPE | 2 |
| & SMP VOL | 7 |
| FILTER POS | 5 WL 550 |
| DELAY | 5 00 |
| % RGT VOL | 70 |
| UNITS | 4 mmol/l |
| UNIT FAC | 1·0000 |
| DECIMAL PT | 1 |
| RBL LOW | 0·000 |
| RBL HI | 0·300 |
| RANGE LOW | 0 |
| RANGE HI | 4·84 |
| CAL FACTOR | ..... |
| STD VAL | 1·29 |
| NORMAL LOW | 0·80 |
| NORMAL HI | 1·55 |
| SLOPE | 1·000 |
| INTERCEPT | 0·0000 |
| EP LIM | 0·0100 |

*UV method*

Unreduced phosphomolybdate complex, resulting from the reaction of inorganic phosphates with molybdate ions in acid medium, is determined at 340 nm [11]. The reagent kit used was from Chemetron Chimica SpA, Milan, Italy. The instrument settings were as follows:

0142–0453/89 $3.00 © 1989 Taylor & Francis Ltd.

*Table 1. Precision and accuracy*

| | Within-run ($n$ = 15, each) | | | Between-day ($n$ = 15, each) | | |
|---|---|---|---|---|---|---|
| | Enzymatic colorimetric method | UV method | Molybdenum blue method | Enzymatic colorimetric method | UV method | Molybdenum blue method |
| *Control Serum A* | | | | | | |
| True (assigned) value, mmol/l | 1·00 | 1·00 | 1·00 | 1·00 | 1·00 | 1·00 |
| Value obtained: | | | | | | |
| Mean ($\bar{x}$), mmol/l | 1·01 | 1·02 | 1·06 | 0·99 | 0·98 | 1·04 |
| Standard deviation (SD), mmol/l | 0·003 | 0·013 | 0·016 | 0·009 | 0·019 | 0·016 |
| Coefficient of variation (CV), % | 0·30 | 1·27 | 1·51 | 0·99 | 1·94 | 1·54 |
| Coefficient of precision[1] (CP), % | 99·70 | 98·73 | 98·49 | 99·01 | 98·06 | 98·46 |
| Coefficient of accuracy[2] (CAc), % | 99·00 | 98·00 | 94·00 | 99·00 | 98·00 | 96·00 |
| Coefficient of analysis[3] (CA), % | 98·96 | 97·63 | 93·82 | 98·59 | 97·21 | 95·71 |
| *Control Serum B* | | | | | | |
| True (assigned) value, mmol/l | 2·20 | 2·20 | 2·20 | 2·20 | 2·20 | 2·20 |
| Obtained value: | | | | | | |
| Mean ($\bar{x}$), mmol/l | 2·14 | 2·12 | 2·02 | 2·12 | 2·10 | 2·01 |
| Standard deviation (SD), mmol/l | 0·010 | 0·010 | 0·010 | 0·039 | 0·032 | 0·048 |
| Coefficient of variation (CV), % | 0·47 | 0·47 | 0·50 | 1·84 | 1·52 | 2·39 |
| Coefficient of precision[1], (CP), % | 99·53 | 99·53 | 99·50 | 98·16 | 98·48 | 97·61 |
| Coefficient of accuracy[2], (CAc), % | 97·27 | 96·36 | 91·82 | 96·36 | 95·46 | 91·36 |
| Coefficient of analysis[3], (CA), % | 97·23 | 98·08 | 91·81 | 95·92 | 95·21 | 91·04 |

[1] CP = 100 − CV.

[2] CAc = 100 − CB, where CB is the coefficient of bias defined as [(assigned value − $\bar{x}$)/assigned value] × 100.

[3] CA = 100 − $R$, where $R$ is calculated as $\sqrt{(CV)^2 + (CB)^2}$.

*Table 2. Linearity*

| Standard inorganic phosphorus mmol/l | Inorganic phosphorus found: Enzymatic colorimetric method mmol/l | % | UV method mmol/l | % | Molybdenum blue mmol/l | % |
|---|---|---|---|---|---|---|
| 0·40 | 0·43 | 107·5 | 0·39 | 97·5 | 0·33 | 82·5 |
| 0·80 | 0·83 | 103·8 | 0·75 | 93·8 | 0·70 | 87·5 |
| 1·61 | 1·65 | 102·5 | 1·55 | 96·3 | 1·48 | 91·9 |
| 2·42 | 2·43 | 100·4 | 2·34 | 96·7 | 2·26 | 93·4 |
| 3·23 | 3·18 | 98·4 | 3·11 | 96·3 | 3·12 | 96·6 |
| 4·04 | 4·14 | 102·5 | 4·01 | 99·2 | 4·01 | 99·2 |
| 4·84 | 4·81 | 99·4 | 4·76 | 98·3 | 5·21 | 107·6 |
| 6·46 | 6·07 | 94·0 | 6·27 | 97·0 | 6·26 | 96·9 |

| | |
|---|---|
| CHEM # | |
| NAME | 11 I.PH |
| TYPE | 2 |
| & SMP VOL | 10 |
| FILTER POS | 1 WL 340 |
| DELAY | 1 00 |
| % RGT VOL | 70 |
| UNITS | 4 mmol/l |
| UNIT FAC | 1·0000 |
| DECIMAL PT | 1 |
| RBL LOW | 0·000 |
| RBL HI | 0·275 |
| RANGE LOW | 0 |
| RANGE HI | 4·84 |
| CAL FACTOR | ..... |
| STD VAL | 1·29 |
| NORMAL LOW | 0·80 |
| NORMAL HI | 1·55 |
| SLOPE | 1·000 |
| INTERCEPT | 0·0000 |
| EP LIM | 0·0100 |

*Molybdenum blue method*

Phosphomolybdate complex is reduced to molybdenum blue by iron(II) sulphate [12]. The reagent kit used from Wako Chemicals GmbH, Newss, FR Germany. The instrument settings were as follows:

| | |
|---|---|
| CHEM # | |
| NAME | 11 I.PH |
| TYPE | 2 |
| & SMP VOL | 14 |

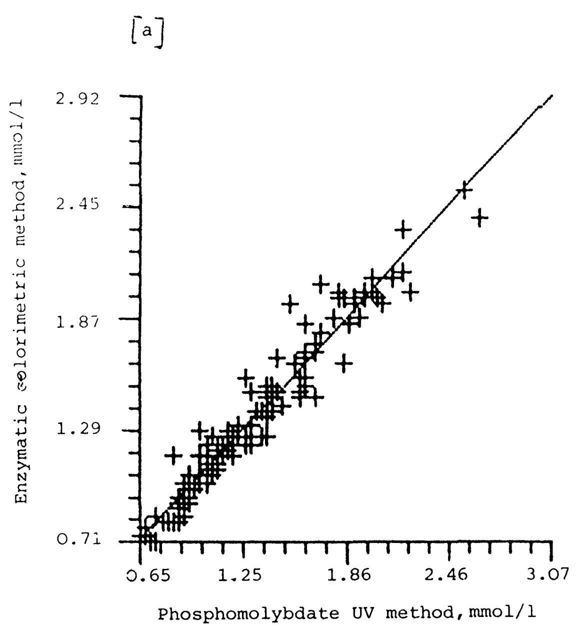

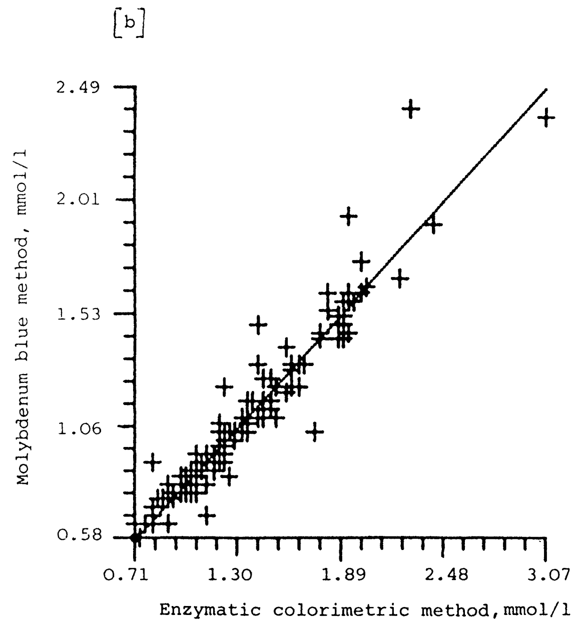

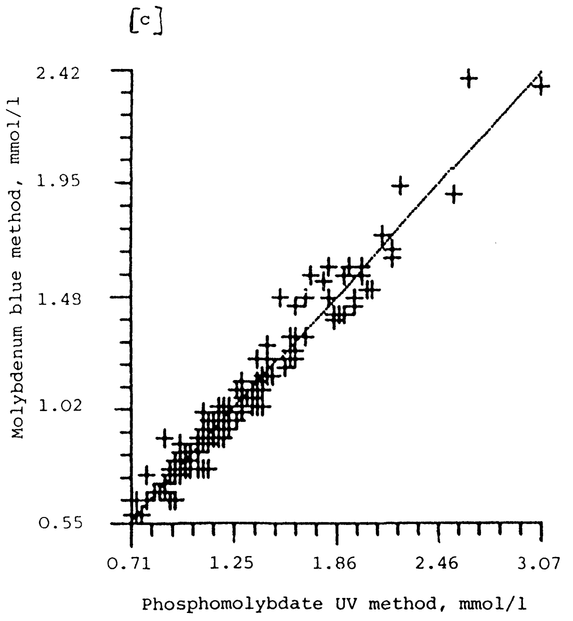

Figure 1. Correlation scattergrams and linear regression parameters for: (a) enzymatic colorimetric method ($y$) vs phosphomolybdate UV method ($x$), where $y = 0{\cdot}898x + 0{\cdot}14$; $r = 0{\cdot}972$; $n = 150$; (b) molybdenum blue method ($y$) vs enzymatic colorimetric method ($x$), where $y = 0{\cdot}808x + 0{\cdot}006$; $r = 0{\cdot}958$; $n = 150$; and (c) molybdenum blue method ($y$) vs phosphomolybdate UV method ($x$), where $y = 0{\cdot}757x + 0{\cdot}08$; $r = 0{\cdot}956$; $n = 150$.

| | |
|---|---|
| FILTER POS | 6 WL 600 |
| DELAY | 5 00 |
| % RGT VOL | 70 |
| UNITS | 4 mmol/l |
| UNIT FAC | 1·0000 |
| DECIMAL PT | 1 |
| RBL LOW | 0·000 |
| RBL HI | 0·250 |
| RANGE LOW | 0 |
| RANGE HI | 4·84 |
| CAL FACTOR | ..... |
| STD VAL | 1·29 |
| NORMAL LOW | 0·80 |
| NORMAL HI | 1·55 |
| SLOPE | 1·000 |
| INTERCEPT | 0·0000 |
| EP LIM | 0·0100 |

### Results

We determined the accuracy and precision of the assays by replicate analysis of two control sera at normal and high phosphate concentrations. These sera were determined by the three methods in 15 replicates for the within-run precision and in single over a 15-day period for the between-day precision. The data obtained were processed statistically according to Louderback and Szatkowski [13]. The coefficients of precision, accuracy and analysis are shown in table 1.

The linearity of the three methods was determined using eight aqueous phosphate solutions ranging from 0·40 mmol/l of inorganic phosphorus. The results are given in table 2.

We explored the effect of potential interferents such as bilirubin and haemoglobin by spiking two pooled human sera at normal (1·20 mmol/l) and elevated (2·40 mmol/l) inorganic phosphorus concentrations with known amounts of these substances. No interference was found for bilirubin up to 175 mg/l with all methods and for haemoglobin up to 750 mg/l with the molybdenum blue method and up to 1000 mg/l with the other two methods.

We found for all the three methods an overestimate of inorganic phosphorus values with lipaemic samples at relatively low triglycerides concentration (about 3500 mg/l); therefore, slightly lipaemic samples should be better analyzed after a pretreatment with a clearing agent.

Finally, we carried out a comparison study to correlate the enzymatic colorimetric method and the two chemical methods. Routine sera (150) were determined in parallel in various runs over 15 days; the samples were selected and stored at −20 °C, and then brought to room temperature prior to determination. Results were processed by least-squares regression analysis; correlation plots and statistical parameters are shown in figure 1.

### Discussion and conclusions

All three methods for determining serum inorganic phosphorus showed high precision. The enzymatic colorimetric method yielded the highest coefficients of precision both for within-run precision (99·7 and 99·5% for control sera A and B, respectively) and between-day precision (99·0 and 98·2%); 1–2% lower results were obtained by the UV and molybdenum blue methods.

As far as accuracy is concerned, the highest coefficient was obtained with the enzymatic colorimetric method, with the UV method yielding results slightly lower (1–2%). Lower (5% on average) results were obtained with the molybdenum blue method, probably because absorbance readings were not made at the peak wavelength of the molybdenum blue dye (i.e. at 600 nm instead of at 650–730 nm) owing to the lack of the appropriate filter.

The linearity extended up to at least 4·84 mmol/l for both the enzymatic colorimetric method and the two chemical methods; this value is approximately four times the upper limit of the reference interval.

The linearity extended up to at least 4·84 mmol/l for both the enzymatic colorimetric method and the two chemical methods; this value is approximately four times the upper limit of the reference interval. The average recovery with respect to the standard values was 101·1 ± 4·0% (SD) for the enzymatic colorimetric method, 96·9 ± 1·6% for the UV method and 94·4 ± 7·6% for the molybdenum blue method; by excluding the highest and the lowest values, the above values are 101·2 ± 2·1%, 96·8 ± 1·9%, and 96·0 ± 6·9%, respectively. Hence the highest correspondence between expected and found concentrations of inorganic phosphorus was found for the enzymatic colorimetric method and the lowest for the molybdenum blue method.

The correlation among methods was good ($r \geq 0{\cdot}956$). The enzymatic method and the UV method agreed very closely, giving comparable results.

In conclusion, the fully enzymatic method for serum inorganic phosphorus determination, which is based on the specific system purine nucleoside phosphorylase/xanthine oxidase coupled to an indicator colorimetric reaction similar to the well known Trinder reaction, proved to be very precise and accurate when applied to the Technicon RA-1000 analyzer, by virtue also of the simplicity of the assay procedure. Good results were also obtained with the method based on the direct measurement of the phosphomolybdate complex in the ultraviolet region. Finally, the method involving chemical reduction of the phosphomolybdate complex to molybdenum blue showed lower performance characteristics.

### References

1. Henry, J. B., *Clinical Diagnosis and Management by Laboratory Methods*, (W. B. Saunders, Philadelphia, 1984), p. 145.
2. Tietz, N. W., in *Textbook of Clinical Chemistry*, (W. B. Saunders, Philadelphia, 1986), p. 1317.
3. Shulz, D. W., Passonneau, J. V. and Lowry, O. H., *Analytical Biochemistry*, **19** (1967), 300.
4. Hwang, W. I. and Cha, S., *Analytical Biochemistry*, **55** (1973), 379.
5. Pesce, M. A., Boudourian, S. H. and Nicholson, J. F., *Clinical Chemistry*, **20** (1974), 332.
6. Scopes, R. K., *Analytical Biochemistry*, **49** (1982), 88.
7. Machida, Y. and Nakanishi, T., *Agriculture Biological Chemistry*, **43** (1982), 807.
8. Ravazzani, V., *LAB, Journal of Laboratory Medicine*, **4** (1986), 219.
9. Berti, G., Fossati, P., Melzi D'Eril, G. V., Tarenghi, G. and Musitelli, C., *Clinical Chemistry*, **33** (1987), 312.
10. Berti, G., Fossati, P., Tarenghi, G., Musitelli, C. and Melzi D'Eril, G. V., *Journal of Clinical Chemistry and Clinical Biochemistry*, **26** (1988), 399.
11. Daly, J. and Ertingshausen, G., *Clinical Chemistry*, **18** (1972), 263.
12. Goldenberg, H. and Fernandez, A., *Clinical Chemistry*, **12** (1966), 871.
13. Louderback, A. L. and Szatkowski, P. R., *Clinical Chemistry*, **26** (1980), 774.

